# Acetylation of WRN Protein Regulates Its Stability by Inhibiting Ubiquitination

**DOI:** 10.1371/journal.pone.0010341

**Published:** 2010-04-23

**Authors:** Kai Li, Rui Wang, Enerlyn Lozada, Wei Fan, David K. Orren, Jianyuan Luo

**Affiliations:** 1 Department of Cancer Biology and the Cancer Center, University of Massachusetts Medical School, Worcester, Massachusetts, United States of America; 2 Graduate Center for Toxicology, University of Kentucky, Lexington, Kentucky, United States of America; University of Minnesota, United States of America

## Abstract

**Background:**

WRN is a multi-functional protein involving DNA replication, recombination and repair. WRN acetylation has been demonstrated playing an important role in response to DNA damage. We previously found that WRN acetylation can regulate its enzymatic activities and nuclear distribution.

**Methodology/Principal Finding:**

Here, we investigated the factors involved in WRN acetylation and found that CBP and p300 are the only major acetyltransferases for WRN acetylation. We further identified 6 lysine residues in WRN that are subject to acetylation. Interestingly, WRN acetylation can increase its protein stability. SIRT1-mediated deacetylation of WRN reverses this effect. CBP dramatically increases the half-life of wild type WRN, while mutation of these 6 lysine residues (WRN-6KR) abrogates this increase. We further found that WRN stability is regulated by the ubiquitination pathway and WRN acetylation by CBP significantly reduces its ubiquitination. Importantly, we found that WRN is strongly acetylated and stabilized in response to mitomycin C (MMC) treatment. H1299 cells stably expressing WRN-6KR, which mimics unacetylated WRN, display significantly higher MMC sensitivity compared with the cells expressing wild-type WRN.

**Conclusion/Significance:**

Taken together, these data demonstrate that WRN acetylation regulates its stability and has significant implications regarding the role of acetylation on WRN function in response to DNA damage.

## Introduction

Mutations in the *WRN* gene cause Werner syndrome (WS), a rare autosomal recessive disorder. WS is a cancer prone syndrome and also displays premature aging [1]. In culture, WS cells undergo replicative senescence much more rapidly than normal cells do, and are also hyper-sensitive to several DNA damaging agents [2]–[5]. In addition, WS cells show increased genomic instability [6], [7]. WRN is a 1432-amino-acid (160 kD) protein and a member of human RECQ helicase family [8]. There are five known members in this family, RecQ1, BLM, WRN, RecQ4 and RecQ5. Mutations in BLM and RecQ4 also cause Bloom Syndrome [9], and Rothmund Thomason syndrome [10], respectively. Consistent with other family members, WRN protein contains 3′ to 5′ helicase activity and DNA-dependent ATPase activity. However, among the human RecQ family members, WRN protein uniquely contains 3′–5′ exonuclease activity [11]. WRN appears to be a multi-functional component in various processes, including DNA replication, recombination, repair, and telomere maintenance, possibly through interacting with different proteins [12].

Posttranslational modification can regulate protein-protein interaction, modulate enzymatic activities, and influence cellular localization and protein stability [13]. WRN can be acetylated in response to various DNA damaging agents [14]–[17]. WRN acetylation facilitates its translocation from nucleoli to nucleoplasm [14], [16] and also regulates its enzymatic activities [16], [17]. These findings indicate that WRN acetylation plays an important role in the cellular response to DNA damage.

Acetylation can regulate the stability of various proteins. Acetylation of the C-terminal domain of p53 is sufficient to abrogate its ubiquitination and extend its half-life *in vivo* [18]. SREBPs were reported to be acetylated by CBP/p300, and acetylation stabilizes members of the SREBP family of transcription factors [19]. Acetylation of hepatocyte nuclear factor 6 (HNF6) results in increasing both HNF6 protein stability and its ability to stimulate transcription of the glucose transporter 2 promoter [20]. Acetylation of β-catenin by PCAF can up-regulate its abundance by inhibiting its ubiquitination and improving its stability [21]. E2F1 can also be acetylated by PCAF, a requirement to stabilize the protein in response to DNA damage [22]. Acetylation of Smad7 by p300 can stabilize and protect it from TGFβ-induced degradation. The same lysines in Smad7 subject to acetylation are also targeted by ubiquitination. Importantly, acetylation of Smad7 can prevent its ubiquitination [23].

Recently, Kahyo and colleagues reported that sirtuin-mediated deacetylation can stabilize WRN protein [24]. In order to further understand the regulation of WRN acetylation and its stability, we identified WRN acetylation sites and made a WRN mutant that could not be acetylated to test its stability. We found that WRN acetylation can increase its protein stability. Deacetylation of WRN by SIRT1 can reverse this effect. CBP dramatically increases the half-life of wild type WRN, while this increase is abrogated with the WRN acetylation mutant. We further found that WRN stability is regulated by the ubiquitination pathway, and WRN acetylation by CBP can dramatically reduce its ubiquitination. These findings advance our understanding on WRN regulation in response to DNA damage.

## Results

### Identification of WRN acetylation sites

**Figure 1:**
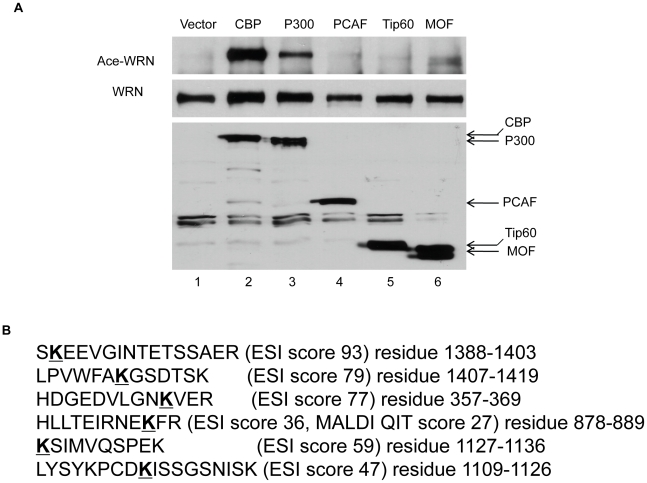
Identification of WRN acetylation sites. **A**, acetylation assay in cells with different acetyltransferases. Western blot analysis was performed with anti-acetylated lysine antibody (Upper panel) or with anti-WRN antibody. The experiment was repeated at least two times. **B**, mass-spectrometry analyses identify the WRN acetylation sites. Acetylated WRN protein was purified by co-transfection of FLAG-tagged WRN with both CBP and p300. The protein was digested with trypsin and analyzed by both MALDI-TOF and LC-ESI MS/MS. Scores of ESI or MALDI QIT were shown. Mascot scores greater than 40 or found in both MALDI and ESI were most confident for the true detection of acetylation.

Different acetyltransferases may acetylate different sites of their target proteins. In order to identify WRN acetylation sites, we first screened several acetyltransferases associated with histone protein acetylation including CBP/p300, PCAF, MOF, and Tip60 for WRN acetylation. After co-transfection of FLAG-tagged WRN with each of the acetyltransferases into HEK293 cells, WRN protein was immunoprecipitated and its acetylation level was detected by Western blot with anti-acetylated lysine antibody. As shown in Fig. 1A, WRN acetylation is clearly detected with CBP (lane 2) and p300 (lane 3) co-transfection, but not with PCAF, MOF, and Tip60 co-transfection (lanes 4–6), indicating that CBP and p300 are the major acetyltransferase for WRN acetylation.

We then purified acetylated WRN protein after co-transfection of FLAG-tagged WRN with both CBP and p300. The protein was digested with trypsin and analyzed by both MALDI-TOF and LC-ESI MS/MS. A number of acetylated lysine containing peptides were identified (Fig. 1B). The acetylated lysine residues in WRN were K366, K887, K1117, K1127, K1389, and K1413.

### Confirmation of WRN acetylation sites in cells

**Figure 2:**
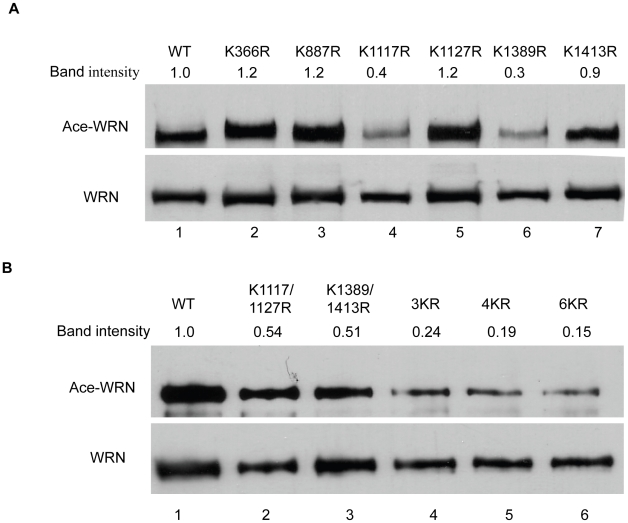
Confirmation of WRN acetylation sites in cells using acetylation assay. **A**, WRN-WT or single mutant was transfected with CBP into HEK293 cells. After immunoprecipitation with FLAG M2 beads, the IP products were subjected to SDS-PAGE and Western blotting with anti-acetylated lysine and anti-WRN antibodies. **B**, WRN-WT or double mutant or triple mutant or quadruple mutant or 6KR mutant was transfected with CBP into cells. The same assay described in A was performed. All the experiments were repeated at least three times. The bands of WRN proteins were quantified by ImageJ software.

We used the in cell acetylation assay to confirm the WRN acetylation sites identified by mass spectrometry analysis. Our strategy was to mutate these putatively acetylated lysines to arginines, since arginine can not be acetylated but retains the positive charge. First, single mutations were made on all 6 lysines. Single mutations of K1117R and K1389R showed significant reductions in the overall level of WRN acetylation (Fig. 2A, lanes 4 and 6). Then, the double mutation constructs K1117/1127R and K1389/1413R were constructed. Both double mutants showed decreased WRN acetylation (Fig. 2B, lanes 2 and 3). Furthermore, upon expression of the triple mutant K1127/1389/1413R and quadruple mutant K1117/1127/1389/1413R, both mutants showed further decreased WRN acetylation level but weak acetylation still can be detected (Fig. 2B, lanes 4 and 5). We finally mutated all of these six lysines to arginines. WRN acetylation was at the lowest level detected in this WRN-6KR mutant (Fig. 2C, lane 6), indicating that all 6 lysines are subject to WRN acetylation.

### CBP stabilizes WRN while SIRT1 reverses this effect

**Figure 3:**
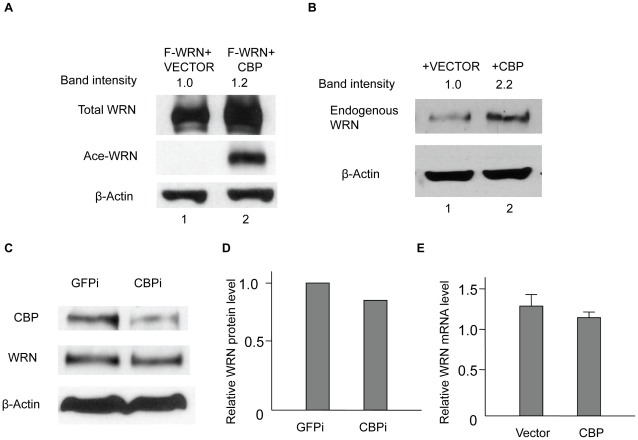
CBP stabilizes WRN protein. **A**, Flag-WRN alone or with CBP was transfected into HEK293 cells. After immunoprecipitation with FLAG M2 beads, the IP products were subjected to SDS-PAGE and Western blotting with anti-WRN and anti-acetylated lysine antibodies. **B**, endogenous WRN protein level was increased by CBP. Vector or CBP were transfected into HEK293 cells. Cell lysates were analyzed by Western blot with anti-WRN and anti-β-actin antibodies. **C**, endogenous WRN protein level was decreased by CBP knockdown. HEK293 cells were transfected with siRNA for CBP or GFP. Cell lysates were analyzed by Western blot with anti-WRN, anti-CBP, and anti-β-actin antibodies. **D**, WRN protein levels from **C** were quantified. **E**, Vector or CBP was transfected into HEK293 cells. Total RNA was isolated and cDNA were synthesized using the Superscript III enzyme. Real-time PCR was performed to detect the WRN mRNA level. All the experiments were repeated at least three times. The bands of WRN proteins were quantified by ImageJ software.

When acetylation assays were performed, we always noticed that co-transfection of WRN and CBP not only resulted in WRN acetylation (Fig. 3A, lane 2, lower panel), but also led to a significant increase in the level of WRN protein (Fig. 3A, lane 2 vs. lane 1, upper panel). We further investigated whether CBP can stabilize the endogenous WRN level by overexpressing CBP in HEK293 cells; indeed, endogenous WRN was increased by CBP overexpression (Fig. 3B, lane 2 vs. lane 1). We also down-regulated CBP by RNAi and examined its effect on WRN. Our results show that WRN protein level was slightly reduced after CBP knockdown (Fig. 3C and D). Since CBP is also a transcriptional co-activator, it is possible that CBP may increase WRN gene expression. We examined the WRN RNA level by Real-time PCR in HEK293 cells transfected with CBP or empty vector. When RNA was isolated 12 hours after transfection, WRN mRNA levels did not significantly change (Fig. 3E), indicating that the observed increase in WRN protein is the result of a CBP-mediated posttranslational event.

**Figure 4:**
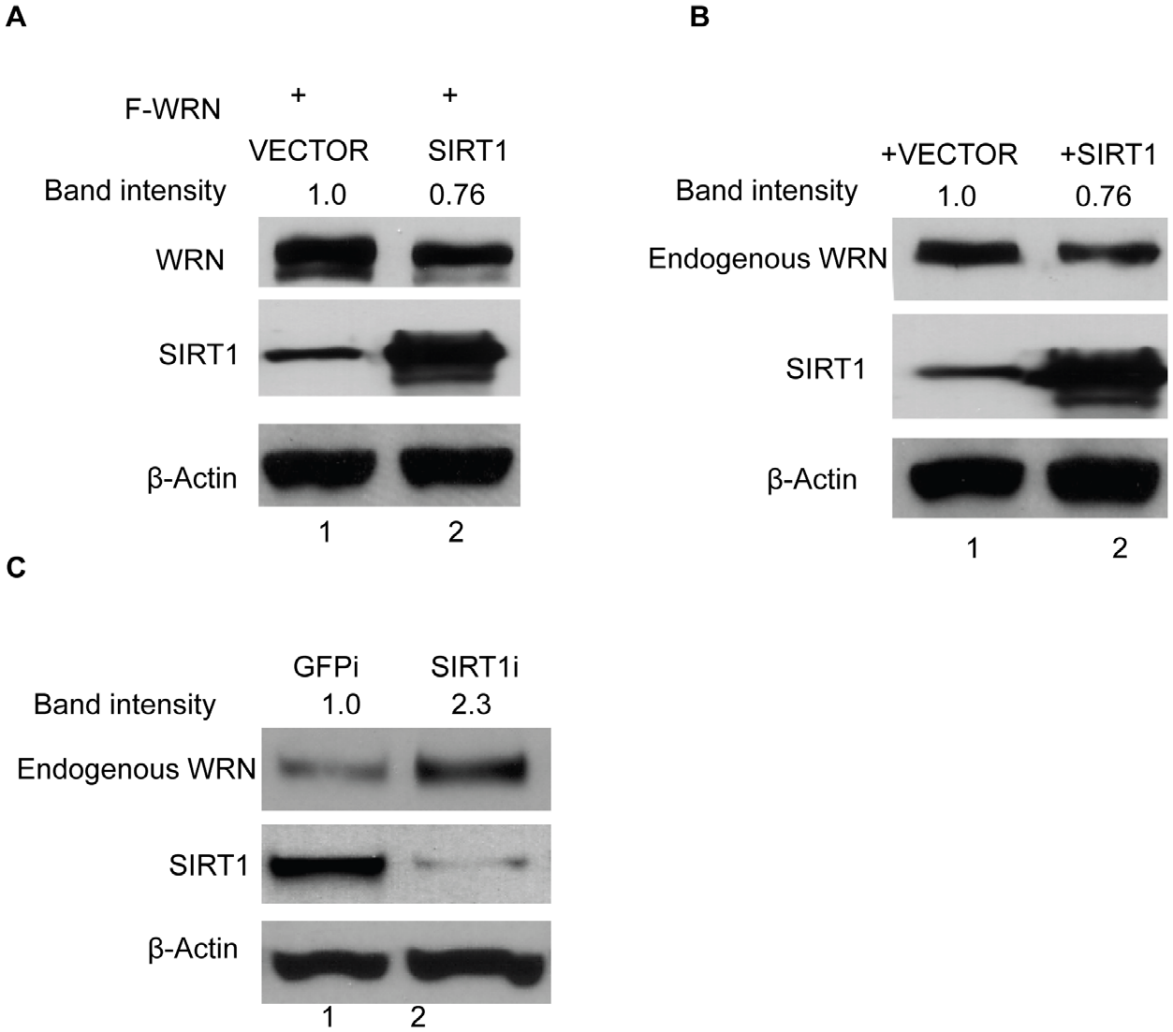
SIRT1 destabilizes WRN protein. **A**, Flag-WRN alone or with SIRT1 was transfected into HEK293 cells. Cell lysates were subjected to SDS-PAGE and Western blotting with anti-WRN, anti-SIRT1 and anti-β-Actin antibodies. **B**, endogenous WRN protein level was decreased by SIRT1. Vector or SIRT1 was transfected into HEK293 cells. Cell lysates were analyzed by Western blot with anti-WRN, anti-SIRT1, and anti-β-actin antibodies. **C**, endogenous WRN protein level was increased by SIRT1 knockdown. HEK293 cells were transfected with siRNA for SIRT1 or GFP. Cell lysates were analyzed by Western blot with anti-WRN, anti-SIRT1, and anti-β-actin antibodies. All the experiments were repeated at least three times. The bands of WRN proteins were quantified by ImageJ software.

We previously found that the NAD-dependent histone deacetylase SIRT1 can specifically deacetylate WRN and regulate its enzymatic activities and cellular localization [16]. Here, we also examined if SIRT1 can reverse CBP stabilization of WRN. Flag-WRN and SIRT1 (or vectors alone) were co-transfected into HEK293 cells and the level of WRN was detected by Western blotting. SIRT1 overexpression clearly decreases WRN protein level (Fig. 4A, lane 2 vs. lane 1, upper panel). We further examined endogenous WRN protein level by SIRT1 expression. As expected, overexpression of SIRT1 in cells also reduced the level of endogenous WRN (Fig. 4B, lane 2 vs. lane 1, upper panel). We then knocked down endogenous SIRT1 by RNAi in the cells (Fig. 4C, lane 2, middle panel); endogenous WRN levels were clearly increased (Fig. 4C, lane 2 vs. lane 1, upper panel). These results indicate that WRN protein is stabilized through CBP-mediated acetylation, and SIRT1 can reverse this effect by deacetylation of WRN.

### WRN acetylation mutant is unable to be stabilized by CBP

**Figure 5:**
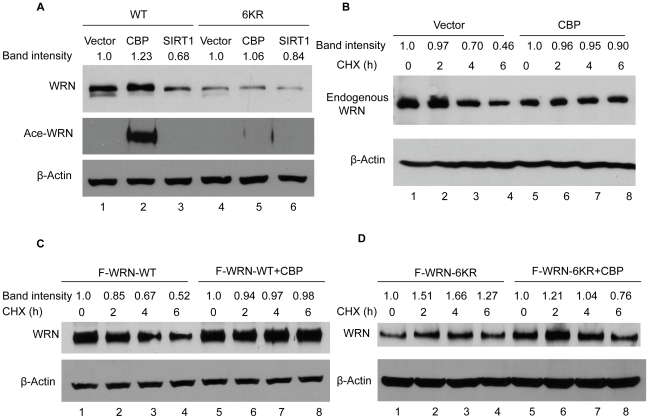
CBP stabilizes WRN protein through acetylation. **A**, WRN-WT or WRN-6KR were transfected with vector, CBP or SIRT1 into HEK293 cells. Cell lysates were analyzed by Western blot with anti-FLAG and anti-β-actin antibodies. WRN acetylation levels were detected by anti-acetylated lysine antibody. **B**, CBP increased endogenous WRN half-life. Vector or CBP were transfected into HEK293 cells. Cells were treated with cycloheximide for the indicated time. Cell lysates were analyzed by Western blot with anti-WRN and anti-β-actin antibodies. **C**, Flag-WRN-WT alone or with CBP was transfected into HEK293 cells. Cells were treated with cycloheximide for the indicated time. Cell lysates were analyzed by Western blot with anti-FLAG and anti-β-actin antibodies. **D**, CBP was unable to increase WRN-6KR half-life. Flag-WRN-6KR alone or with CBP was transfected into HEK293 cells. Cells were treated with cycloheximide for the indicated time. Cell lysates were analyzed by Western blot with anti-FLAG and anti-β-actin antibodies. All the experiments were repeated at least three times. The bands of WRN proteins were quantified by ImageJ software.

Our finding that CBP stabilizes WRN through acetylation has been further examined by expressing WRN acetylation mutant in cells. Vectors expressing FLAG-WRN-wt or FLAG-WRN-6KR were co-transfected with empty vector, CBP- or SIRT1-expression vector into HEK293 cells and the levels of WRN protein were detected by Western blotting. As expected, CBP increases WRN-wt levels, while SIRT1 reverses this effect (Fig. 5A, lanes 1–3, upper panel). In contrast, CBP failed to increase the level of mutant WRN-6KR protein (Fig. 5A, lane 5 vs. lane 4, upper panel), while SIRT1 co-transfection only showed a slight decrease in WRN-6KR protein levels (Fig. 5A, lane 6 vs. lane 4, upper panel), indicating that the acetylation mutant abrogated the ability of CBP to stabilize WRN. We further examined if CBP can affect the half-life of WRN. Vector or CBP were transfected into HEK293 cells. Cells were treated with cycloheximide for the indicated time and the levels of endogenous WRN protein were detected by Western blotting. CBP indeed increased the half-life of endogenous WRN (Fig. 5B, lanes 5–8 vs. lanes 1–4, upper panel). We then examined the effect of CBP on wild type WRN and acetylation mutant 6KR. FLAG-WRN-wt or 6KR alone or with CBP were transfected into HEK293 cells. The same assay was performed to examine the half-life of WRN protein. CBP increased the half-life of WRN-wt (Fig. 5C, lanes 5–8 vs. lanes 1–4, upper panel) but failed to increase that of WRN-6KR (Fig. 5D, lanes 5–8 vs. lanes 1–4, upper panel). All these data demonstrate that CBP stabilizes WRN protein through acetylation.

### Acetylation stabilizes WRN protein through inhibiting its ubiquitination

**Figure 6:**
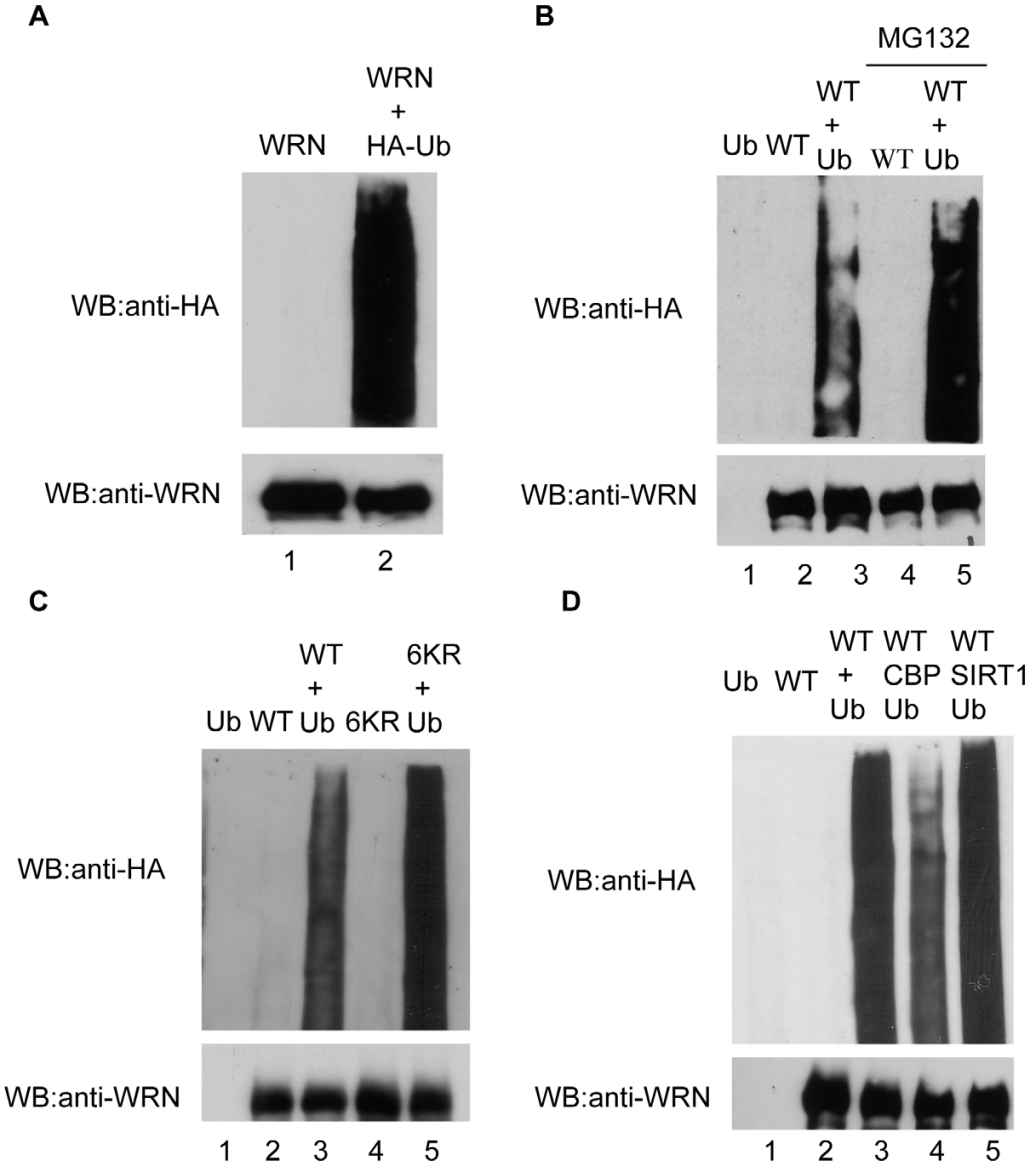
WRN acetylation inhibits its ubiquitination. **A**, Flag-WRN alone or with HA-ub was transfected into HEK293 cells. After immunoprecipitation with FLAG M2 beads, the IP products were subjected to SDS-PAGE and Western blot with anti-HA and anti-WRN antibodies. **B**, Flag-WRN alone or with HA-ub was transfected into HEK293 cells. Cells were treated with or without MG132 (5 µM, 8 hr). The same assay described in A was performed. **C**, FLAG-WRN-WT or 6KR was transfected with vector, HA-ub into HEK293 cells. The same assay described in A was performed. **D**, FLAG-WRN was transfected with vector, HA-ub, CBP and HA-ub, or SIRT1 and HA-ub into HEK293 cells. The same assay described in A was performed. All the experiments were repeated at least three times.

WRN has been reported to be regulated by the ubiquitination pathway [24]. We further examined if acetylation stabilizes WRN protein by inhibiting its ubiquitination. FLAG-WRN alone or with HA-Ub were transfected into HEK293 cells. After immunoprecipitation with FLAG M2 beads, the IP products were subjected to SDS-PAGE and Western blotting with anti-HA and anti-WRN antibodies. WRN ubiquitination can be clearly detected (Fig. 6A, lane 2 vs. lane 1) and is strongly enhanced by the proteasome inhibitor MG132 (Fig. 6B, lane 5 vs. lane 3). The acetylation mutant WRN-6KR was also examined by this ubiquitination assay. FLAG-WRN-wt or –WRN-6KR was transfected with vector or HA-Ub into HEK293 cells. WRN-6KR showed stronger ubiquitination than WRN-wt (Fig. 6C, lane 5 vs. lane 3), indicating that WRN acetylation and ubiquitination do not compete for the same set of lysine residues.

The effect of CBP and SIRT1 on WRN ubiquitination was further investigated. FLAG-WRN was co-transfected with vector, HA-Ub, CBP and HA-Ub, or SIRT1 and HA-Ub into HEK293 cells. Co-transfection of CBP clearly decreased WRN ubiquitination (Fig. 6D, lane 4 vs. lane 3), while SIRT1 showed the opposite effect (Fig. 6D, lane 5 vs. lane 4). These results suggest that CBP and SIRT1 regulate WRN ubiquitination through acetylation/deacetylation.

### WRN protein is acetylated and stabilized in response to MMC

**Figure 7:**
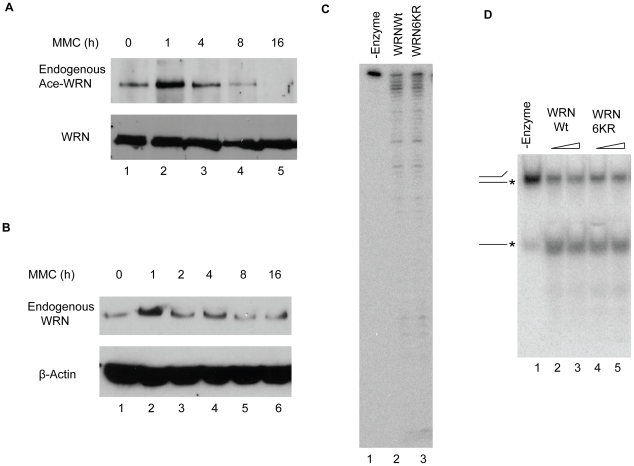
WRN is acetylated and stabilized in response to MMC treatment. **A**, HEK293 cells were treated with 0.5 µg/ml MMC for indicated time. Cell lysates were immunoprecipitated by anti-acetylated WRN-K1389 antibody and western blotted by anti-WRN antibody (upper panel). Portions of the cell lysates were western blotted by anti-WRN antibody to determine the equal amount of WRN protein in lysates for anti-acetylated WRN antibody immunoprecipitation shown in upper panel. **B**, HEK293 cells were treated with 0.5 µg/ml MMC for indicated time. Equal amounts of total protein were loaded on each lane and western blotted by anti-WRN antibody to determine the WRN amount (upper panel). Anti-β-Actin blot was used as loading control (lower panel). **C**, Equal amounts of purified WRN-wt and WRN-6KR mutant were assayed for exonuclease activity on a two-stranded fork substrate as described in Methods and DNA products were analyzed after denaturing PAGE. **D**, Equal and increasing amounts of purified WRN-wt and WRN-6KR mutant proteins were assayed for helicase activity on a two-stranded fork and the products analyzed by native PAGE as described in Methods. All the experiments were repeated at least two times.

In order to explore the functional consequence of WRN stabilization by acetylation, we treated cells with mitomycin C (MMC), an agent that generates DNA interstrand crosslinks, lesions that WRN function is thought to address [5]. MMC treatment strongly induced WRN acetylation in cells that peaked at one hour post-treatment (Fig. 7A, lane 2). As expected, WRN protein was stabilized at this same time point (Fig. 7B, lane 2).

**Figure 8:**
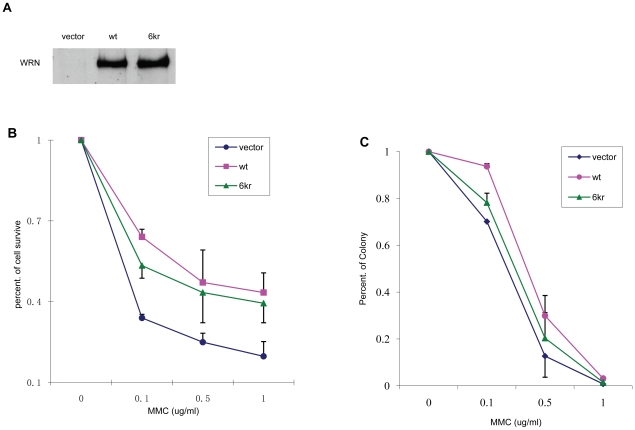
WRN acetylation helps cell survival in response to MMC treatment. **A**, cells from three virus infected stable cell lines H1299/vector (lane 1), H1299/Flag-WRN-WT (lane 2) and H1299/Flag-WRN-6KR (lane3) were lysed and immunoprecipitated by anti-Flag M2 beads. WRN protein expression levels were determined by western blot with anti-WRN antibody. **B**, cells from three stable cell lines were treated with increasing doses of MMC for 1 hr, followed by 48 h recovery. MTT assays were performed as detailed under “Materials and Methods”. **C**, cells from three stable cell lines were treated with increasing doses of MMC for 1 hr, and then allowed to grow for about 2 weeks. The formed colonies were fixed in ice-cold methanol and stained with 0.5% crystal violet solution. The formed colonies were counted. All the experiments were repeated at least three times.

To further investigate whether WRN acetylation could affect cellular response to MMC treatment, we generated three stable transfectant cell lines. H1299 cells were infected with pBabe control virus or virus encoding WRN-wt or WRN-6KR containing lysine to arginine mutations at 6 lysine positions (Fig. 1B) that mimics hypoacetylated WRN. WRN expression levels for the latter two cell lines are similar (Fig. 8A). We also examined both exonuclease and helicase activities for the purified WRN-wt and WRN-6KR mutant. The WRN-6KR mutant retains similar activities as the WRN-wt (Fig. 7C and D), indicating that this mutant protein folds properly and also that the 6 arginine substitutions do not significantly alter WRN's catalytic activities. MTT assay was carried out to detect cell viability of these three WRN cell lines in response to MMC treatment. As shown in Fig. 8B, expression of the WRN-6KR mutant that mimics hypoacetylated WRN resulted in a less efficient rescue of MMC treatment compared to WRN-wt. We also examined colony formation of the three WRN stable lines after MMC treatment. As shown in Fig. 8C, the number of colonies from cells expressing WRN-6KR was significantly reduced compared to WRN-wt cells in response to the same dose of MMC. The vector control cells had the lowest survival rate in both assays. These results indicate that the WRN-6KR acetylation mutant partially impaired WRN function and suggest that acetylation of WRN is physiologically important for cell survival in response to MMC-induced DNA damage.

## Discussion

Our previous study has demonstrated that WRN can be acetylated *in vivo*, and WRN acetylation can regulate its enzymatic activities and nuclear localization [16]. Here, we investigated WRN acetylation in further depth, including how it regulates WRN stability. We identified 6 lysines as major acetylation sites of WRN protein by mass spectrometry analysis and acetylation assays in cells. We further found that WRN acetylation can regulate WRN protein stability by inhibiting its ubiquitination. Importantly, WRN is strongly acetylated and stabilized in response to mitomycin C treatment. H1299 cells stably expressing WRN-6KR that mimics unacetylated WRN display significantly higher MMC sensitivity compared with the cells stably expressing wild-type WRN. These results demonstrate that WRN acetylation plays significant physiological functions in response to DNA damage.

The functions of WRN acetylation have been reported by several groups. It was first reported by Blander and colleagues that WRN acetylation is induced by DNA damage and is associated with translocation from nucleoli to DNA damage foci [14]. Our previous studies confirmed this result and further found that the NAD-dependent histone deacetylase SIRT1 can deacetylate WRN and also participate in the regulation of WRN translocation [16]. Vaitiekunaite and colleagues also found that SIRT1 can regulate WRN translocation [25]. In addition, we found that WRN acetylation can influence its enzymatic activities [16]. Muftuoglu and colleagues also found that acetylation of WRN altered its activity although their findings are somewhat different [17], perhaps due to different DNA substrates or assay methods. Nevertheless, the results indicate that WRN enzymatic activities can be regulated by acetylation in response to DNA damage. WRN acetylation also is suggested to play an important role in regulating base excision DNA repair [17]. In this study, we found that acetylation can stabilize WRN protein through inhibiting its ubiquitination and acetylation of WRN is important for cell survival in response to MMC treatment. These findings contribute significantly to our understanding of how WRN acetylation regulates its functions. With our complete identification of the WRN acetylation sites and production of acetylation mutants, more physiological roles of WRN acetylation will be revealed.

We consistently observe that WRN protein levels increase when we co-transfected WRN with CBP/p300 compared with WRN alone. Since there is no obvious change in WRN mRNA levels (Fig. 3C), this increased WRN level does not occur through transcription activation. CBP/p300 has been reported to regulate the stability of several proteins through acetylation, apparently by inhibiting ubiquitination of the proteins. However, the precise mechanisms could be different. Acetylation-mediated stabilization of Smad7 is through competition between acetylation and ubiquitination of the same lysine residues—i.e., acetylation of these residues prevented subsequent ubiquitination [23]. The same competitive mechanism was reported for acetylation of SREBP1a [19]. On the other hand, acetylation of the p53 C-terminal domain is sufficient to affect its ubiquitination by mdm2 elsewhere on the protein. This indicates that p53 acetylation regulates its ubiquitination through other mechanisms in addition to the site competition [18]. Our results demonstrating an increased amount of ubiquitination in WRN-6KR acetylation mutant (Fig. 6C) supports the notion that acetylation can protect a protein from ubiquitination by other means than direct competition for modification sites. It is of note that PCAF, another acetyltransferase, has also been reported to stabilize E2F1 [22] and β-Catenin [21] through acetylation. Our results show, however, that PCAF is unable to acetylate WRN (Fig. 1A).

Our previous findings showed that SIRT1 can deacetylate WRN and also participate in the regulation of WRN translocation and enzymatic activities [16]. In this study, we examined the role of SIRT1 in the regulation of WRN stability. Overexpression of SIRT1 in cells decreases WRN protein levels, while down regulating SIRT1 by RNAi in cells can increase the WRN level (Fig. 4). Consistent with these findings and those detailed above, co-transfection of SIRT1 with WRN in cells increased the WRN ubiquitination level (Fig. 6D). Our findings seem to be at odds with the results recently reported by Kahyo and colleagues, who found that the sirtuin-mediated deacetylation pathway stabilizes WRN protein. However, their assay uses the SIRT1 inhibitor Sirtinol for treating cells to detect WRN protein levels, without showing WRN acetylation and SIRT1-mediated WRN deacetylation. Thus, it is possible that treating cells with Sirtinol decreased WRN protein levels through other mechanisms. Nevertheless, our results showing that SIRT1 down-regulates WRN protein levels are also supported by recent report by Vaitiekunaite and colleagues, who found that increased SIRT1 expression in cells was associated with down-regulation of endogenous WRN protein levels [25].

WRN is a multi-functional protein potentially involved in many processes in DNA metabolism [26]. How WRN protein is regulated in response to DNA damage is of great interest. Our findings that acetylation of WRN stabilizes the protein through inhibiting its ubiquitination and optimizes the response to MMC treatment advanced our understanding of WRN regulation following DNA damage. The exact mechanism of how WRN acetylation prevents its ubiquitination remains unclear. It seems probable that acetylation of WRN induces a conformational change to prevent its ubiquitination. It is also possible that acetylation of specific lysines in WRN can influence its interaction with other proteins or DNA, that in turn masks other lysines from ubiquitination. Further work is needed to understand the molecular details of these events.

## Materials and Methods

### Culture medium and reagents

HEK293 and H1299 cells were obtained from American Type Culture Collection and cultured in Dulbecco's modified Eagle's medium supplemented with 10% fetal bovine serum. Anti-WRN antibody (H300) was purchased from Santa Cruz Biotechnology (Santa Cruz, CA). Anti-β-actin monoclonal antibody was purchased from Sigma. Anti-acetylated-lysine antibody was purchased from Cell Signaling Technology (Danvers, MA). Anti-HA antibody (12CA5) was purchased from Roche. Transfection of the SIRT1 siRNA (Dharmacon Inc.) was performed using Oligofectamine reagent (Invitrogen) following the manufacturer's instructions with a final oligonucleotide concentration of 20 µM.

### Immunoprecipitation and Detection of WRN acetylation in cells

Immunoprecipitation and acetylation assays were as described previously [27]. Briefly, HEK293 cells were transfected with FLAG-WRN alone, or with CBP-containing plasmid DNA by the calcium phosphate method. 36 h after transfection, cells were harvested and lysed in FLAG lysis buffer (50 mM Tris, 137 mM NaCl, 1 mM NaF, 1 mM NaVO_3_, 1% Triton X-100 and 0.2% Sarkosyl, 1 mMDTT, 10% glycerol, pH 7.8) containing fresh protease inhibitors, PMSF, 10 µM TSA and 5 mM nicotinamide. Cell extracts were immunoprecipitated with anti-Flag monoclonal antibody M2 beads (Sigma). After elution with the FLAG peptide, the proteins were resolved by 8% SDS-PAGE gels and analyzed by Western blotting with anti-acetylated Lysine or anti-WRN antibodies.

### Real-time PCR

Vector or CBP-containing plasmid DNAs were transfected into HEK293 cells. 12 h after transfection, RNA was isolated using Rneasy (Qiagen, Valencia CA). cDNA were synthesized using the Superscript III enzyme (Invitrogen). Quantitative real-time PCR analysis was performed using SYBR green reagent (Invitrogen) on a DNA engine opticon 2 cycler (Bio-rad laboratories, Hercules CA). The following primers were used: WRN- Forward, GGA TCA GCA CAG TCA GAA AAT GTT CT; WRN-Reverse, GGA TAG ATT CAG TTT CCT AAG TTC ACC; HPRT-Forward, ATC AGA CTG AAG AGC TAT TGT AAT GA; HPRT-Reverse, TGG CTT ATA TCC AAC ACT TCG TG.

### Ubiquitination assay

FLAG-WRN alone or with HA-Ub was transfected into HEK293 cells. 36 h after transfection, cells were harvested and lysed in FLAG lysis buffer (50 mM Tris, 137 mM NaCl, 1 mM NaF, 1 mM NaVO_3_, 1% Triton X-100 and 0.2% Sarkosyl, 1 mMDTT, 10% glycerol, pH 7.8) containing fresh protease inhibitors and PMSF. Cell extracts were immunoprecipitated with anti-FLAG monoclonal antibody M2 beads (Sigma). After elution with 20 µl 2× SDS loading buffer, proteins were resolved by SDS-PAGE and analyzed by Western blotting with anti-HA antibody to detect ubiquitination, and anti-WRN antibody to detect WRN protein level.

### Measuring the half-life of WRN protein

FLAG-WRN alone or with CBP was transfected into HEK293 cells. 12 h after transfection, cells were washed with PBS, and seeded into 60-mm dishes to normalize for the efficiency of the transfection. On the next day, the cells were treated with cycloheximide (100 µg/ml) for indicated time. Cells were harvested and lysed in 100 µl RIPA buffer (10 mM Tris, 150 nM NaCl, 1% DOC, 0.05% SDS, 1% Triton X-100, pH 8.0). Equal amounts of protein were separated by SDS-PAGE and analyzed by Western blotting with anti-WRN antibody and anti-β-actin antibody as a loading control.

### MTT assay

Cells were seeded in 96-well plates, allowed to attach overnight, then treated with increasing doses of MMC (0.1, 0.5, 1.0 µg/ml) for 1 h and then incubated for another 48 h. Aliquots of 10 µl of MTT (5 mg/ml) were added to each well. After 4 h, the color formed was quantitated by a spectrophotometric plate reader (Berkman, Inc) at 595 nm wavelength after solubilization in 100 µl of DMSO.

### Colony formation assay

Cells (1000 per 6 cm dish) were plated in triplicate. After overnight attachment, cells were treated with three different concentrations of MMC (0.1, 0.5, 1.0 µg/ml) for 1 h and then allowed to grow for about 2 weeks. The formed colonies were fixed in ice-cold methanol and stained with 0.5% crystal violet solution. The formed colonies were counted.

### Helicase and exonuclease assays

Helicase assays were performed on a two-stranded fork substrate containing a 31 and 21 nt 5′ and 3′ single-stranded arms, respectively. This substrate was generated by radiolabeling a 62-mer (5′-CACTGACTCCAGGAACTGGAGGATGCCTAGGTGGCCAGCTGCCGTCCAG-ACTCAGAGGAGTG-3′) with [γ-^32^P]-ATP and T4 polynucleotide kinase followed by annealing with unlabeled, partially complementary 52-mer (5′-CACTCCTCTGAGTCTGGACGGCAGCT- GGCCAAGTGTGAGTGTGAGTGTGAGT-3′). Annealed substrate was gel-purified and eluted into 10 mM Tris pH 8.0, 10 mM NaCl. FLAG-tagged WRN-wt and WRN-6KR proteins were incubated with substrate for 30 min at 37°C in 20 µl of WRN reaction buffer (40 mM Tris-HCl, pH 8.0, 1 mM MgCl_2_, 250 µM ATP, 0.1% NP-40, 100 µg/ml BSA, and 5 mM DTT). Reactions were subsequently incubated with Proteinase K (1 mg/ml), SDS (0.2%) and EDTA (5 mM) for 30 min at 37°C and then stopped by addition of one-sixth volume of loading dyes (30% glycerol, 0.25% bromphenol blue, 0.25% xylene cyanol, and 50 mM EDTA). Samples were subjected to native polyacrylamide (6%) gel electrophoresis (PAGE) in 1X TBE buffer at 100 V for 3 h at room temperature. Gels were vacuum-dried and radioactive DNA was visualized by phosphorimaging. The WRN exonuclease assay utilized a similar two-stranded fork substrate (containing 31 and 21 nt 5′ and 3′ single-stranded arms, respectively), constructed by annealing the labeled 62-mer (as above) to an unlabeled 52-mer (5′-TCACTTGACAAGTGACTGTGAC- CTAGGCATCCTCCAGTTCCTGGAGTCAGTG-3′) then purified as above. WRN-wt and WRN-6KR proteins were preincubated on ice for 5 min in 10 µl of WRN reaction buffer (including 1 mM MnCl_2_ instead of MgCl_2_), and then transferred to 37°C for 20 min. Reactions were stopped by addition of formamide loading buffer (95% formamide, 20 mM EDTA, 0.1% bromphenol blue, and 0.1% xylene cyanol), heated at 90°C and the DNA products separated by denaturing (8%) PAGE. After gel drying, digestion of the labeled strand by the 3′ to 5′ exonuclease activity of WRN proteins was visualized by phosphorimaging.
